# Characteristics of Hybrid Pigments Made from Alizarin Dye on a Mixed Oxide Host

**DOI:** 10.3390/ma12030360

**Published:** 2019-01-24

**Authors:** Anna Marzec, Bolesław Szadkowski, Jacek Rogowski, Waldemar Maniukiewicz, Małgorzata Iwona Szynkowska, Marian Zaborski

**Affiliations:** 1 Institute of Polymer and Dye Technology, Faculty of Chemistry, Lodz University of Technology, Stefanowskiego 12/16, 90-924 Lodz, Poland; boleslaw.szadkowski@edu.p.lodz.pl (B.S.); marian.zaborski@p.lodz.pl (M.Z.); 2 Institute of General and Ecological Chemistry, Faculty of Chemistry, Lodz University of Technology, Zeromskiego 116, 90-924 Lodz, Poland; jacek.rogowski@p.lodz.pl (J.R.); waldemar.maniukiewicz@p.lodz.pl (W.M.); malgorzata.szynkowska@p.lodz.pl (M.I.S.)

**Keywords:** Alizarin, hybrid pigment, aluminum-magnesium hydroxycarbonate, mixed oxide, color stability

## Abstract

This paper describes the fabrication of a new hybrid pigment made from 1,2-dihydroxyanthraquinone (alizarin) on a mixed oxide host (aluminum-magnesium hydroxycarbonate, LH). Various tools were applied to better understand the interactions between the organic (alizarin) and inorganic (LH) components, including ion mass spectroscopy (TOF-SIMS), 27-Aluminm solid-state nuclear magnetic resonance (NMR) spectroscopy, X-ray diffraction (XRD), and thermogravimetric analysis (TGA). TOF-SIMS showed that modification of the LH had been successful and revealed the presence of characteristic ions C_14_H_7_O_4_Mg^+^ and C_14_H_6_O_5_Al^−^, suggesting interactions between the organic chromophore and both metal ions present in the mixed oxide host. Interactions were also observed between Al^3+^ ions and Alizarin molecules in ^27^Al NMR spectra, with a chemical shift detected in the case of the modified LH matrix. Any changes in color following reactions with Mg^2+^ and Al^3+^ ions were observed. Some of the physicochemical properties of alizarin, such as resistance to dissolution and color stability at elevated temperatures, were improved in comparison to the pure dye. This effect can be attributed to strong dye-LH interactions and the effective transformation of alizarin into an insoluble form. Moreover, the pigments exhibited higher thermal resistance and greater color stability in comparison to commercially available alizarin lakes (Alizarin Crimson).

## 1. Introduction

Natural and synthetic dyes are used in a wide range of applications, including in the optics, cosmetics, and food packaging industries [1,2]. However, their uses are limited by both their high solubility and low chemical, thermal, and photo resistance. Natural dyes and their identical synthetic analogues can be transformed into insoluble pigments with enhanced chemical and thermal stability [3]. Natural organic dyes have been used since antiquity to produce organic-inorganic pigments, known as lakes [4]. These can be prepared by the complexation of dye molecules with metallic cations present in an inorganic support material, such as alumina, silica, talc, calcium carbonate, or barium sulfate. One of the oldest known compound dyes is alizarin (1,2-dihydroxyanthraquinone), which is extracted from the roots of the common madder plant (*Rubia tinctorium* L.) [5,6]. From a chemical point of view, alizarin belongs to the anthraquinone class of dyes, with condensed aromatic rings, two carbonyls (at positions 9 and 10), and two hydroxyl groups.

The most common method of obtaining alizarin lakes is by the complexation of 1,2-dihydroxyanthraquinone with alumina. This reaction is based on the formation of a six-membered cyclic chelate, with a hydroxyl group (–OH) substituent at position 1 and the nearest oxygen atom from the carbonyl (C=O) [7,8]. The optical properties and stability (thermal and chemical resistance) of alizarin lakes depend on the types of metal salts applied and on the structure of the metal-dye complex [9]. Numerous methodologies are now available for studying the interactions of organic chromophores with the metal ions present in inorganic carriers, including infrared (IR) [10,11] and Raman [12,13] spectroscopies, spectrophotometry, and fluorimetry [14,15]. Studies based on NMR spectroscopy, most often of ^13^C and ^27^Al MAS NMR spectra, suggest that organic dyes form complexes with clay minerals [16,17]. However, the ^27^Al MAS NMR method is restricted to the analysis of dye interactions involving a limited number of ions and is unsuitable for the study of interactions involving ions such as Mg^2+^ or Zn^2+^.

Alizarin can be immobilized and stabilized on various inorganic bases [18,19]. Perez et al. [20] stabilized several dyes, including alizarin, on an acid matrix, α gamma alumina. The stability of the pigment was greatly enhanced by the presence of coordinative unsaturated sites on the surface of the gamma-alumina. Interactions between the alumina and the organic chromophore were found to determine the color and hue of the pigment. The metal-dye complexes were studied using FT-IR and ^27^Al MAS NMR spectroscopy, as well as colorimetry. Trigueiro et al. [21] synthesized Ti- and Al-pillared montmorillonite, which they modified with carminic acid (CA) and alizarin dye. Interactions were observed between the organic guest and the inorganic host using FT-IR, ^13^C, and ^27^Al solid state magnetic nuclear resonance and time resolved fluorescence spectroscopies. The organic-inorganic pigments based on Al-pillared montmorillonite showed a higher stability under UV irradiation than the Ti-based hybrid. Ghannam et al. [22] synthesized sensitive colored hybrid inorganic/organic pigments based on polymer-coated mica particles. The color variations were attributed to the superimposition of different layers of adsorbed alizarin molecules. When closer to the surface, alizarin is more influenced by oxanions and is pinkish in color. When it is further from the surface, its electronic state is affected less and it has a yellowish hue. The global color may therefore be ascribed to the combined effect of the different electronic states of alizarin, resulting in an orange pigment.

In this study, a novel hybrid pigment was produced by the complexation of 1,2-dihydroxyanthraquinone dye with aluminum-magnesium hydroxycarbonate (LH). In a previous work [23], LH with different Mg:Al ratios had been employed to produce new color-tunable hybrid pigments. In the present study, we used an Al rich mixed oxide (Al:Mg ratio 80:20) with a large surface area to stabilize the alizarin chromophore. Interactions between the alizarin and metal ions in the LH host (Mg^2+^ and Al^3+^) were confirmed by mass spectroscopy (TOF-SIMS). A common method for investigating interactions between organic chromophores and Al ions, ^27^Al solid state magnetic nuclear resonance, was also used to confirm the formation of an alizarin-Al complex. The morphology of the hybrid pigment was characterized by X-ray powder diffraction (XRD) and scanning electron microscopy (SEM). The organic-inorganic hybrid was evaluated for thermal stability (TGA) and resistance to dissolution. The physical and chemical properties of the new alizarin lake were compared to those of a commercially available alizarin-based lake pigment (Alizarin Crimson).

## 2. Materials and Methods

### 2.1. Raw Materials

The inorganic host, magnesium-aluminum hydroxycarbonate with an Mg/Al weight ratio of 20/80, was purchased from Sasol GmbH (Schnelldorf, Germany). Toluene, ethanol, acetone, and cyclohexane were purchased from Sigma-Aldrich (Schnelldorf, Germany). The color agents Alizarin Crimson (madder lake) and alizarin were kindly supplied by Kremer Pigmente GmbH & Co. KG (Munich, Germany) and Sigma-Aldrich (Schnelldorf, Germany), respectively. The reagents were analytical grade and did not require further purification prior to use.

### 2.2. Synthesis of Hybrid Pigments

Pigments modified with alizarin (LH/Alizarin) were prepared in an aquatic environment with high purity deionized water. For LH/Alizarin (15%), the method proceeded as follows: 8.5 g of LH was added dropwise with vigorous stirring to a solution of 1.5 g of alizarin in 200 cm^3^ water and 50 cm^3^ ethanol. During modification, the pH value changed from 9.4 to 8.1. The mixture was then heated to 80 °C under mechanical agitation. The reaction was continued under the same conditions for a further 3 h. Finally, the reaction mixture was filtered, washed with water, and dried at 70 °C until a dry hybrid pigment powder was obtained.

### 2.3. Characterization of Hybrid Pigments

Secondary ion mass spectra of positive and negative ions were obtained using a TOF-SIMS IV mass spectrometer (IONTOF GmbH, Muenster, Germany) equipped with a 25 keV bismuth primary ion gun and a high mass resolution time of flight mass analyzer. The primary ion beam current was set to 0.4 pA. The structural changes in the examined samples were observed using powder X-ray diffraction (PXRD) analysis, with a PANalytical X’Pert Pro MPD diffractometer (Malvern Panalytical Ltd., Royston, UK) in Bragg-Brentano reflection geometry and (CuK_α_) radiation from a sealed tube in the range of 2*θ* = 3°–70° at a step length of 0.0167°. The XRD curves were interpreted based on the positions of the basal reflections. Solid state Nuclear Magnetic Resonance (MAS NMR) measurements were performed in a Bruker Avance III 400 WB spectrometer (Rheinstetten, Germany) operating at a resonance frequency of 104.26 MHz. ^27^Al chemical shifts were compared to AlCl_3_·6H_2_O in 1M solution as an external reference (0 ppm). The thermal stability of the hybrid pigments was determined using a Q500 Thermogravimetric Analyzer, (TA Instruments, Greifensee, Switzerand). Thermogravimetric analysis (TGA) was performed with a heating rate of 10 °C/min under a nitrogen atmosphere, across a temperature range of 25–600 °C. The specific surface area was measured based on nitrogen adsorption at 77 K over a relative pressure (P/P0) range of 10^−6^–1 using a Gemini 2360 V2.01 porosimeter (Micromeritics, Norcross, GA, USA). Measurements were performed according to the Brunauer-Emmett-Teller (BET) nitrogen adsorption method. The samples were degassed for 20 h at 100 °C under a vacuum. The solvent resistance of the hybrid pigments was evaluated based on the PN-C-04406 standard. The pigment samples were immersed in cylinders with solvent (acetone, toluene, or ethyl alcohol) or water for 24 h, at room temperature. Their solvent resistance was estimated (on a scale of 1/5) based on the degree of decolorization. The UV-VIS spectra were recorded using an Evolution 201/220 UV-Visible Spectrophotometer (Thermo Fisher Scientific, Waltham, MA, USA). The experiment was conducted in a spectral window from 1100 to 200 nm at room temperature. The measurement specimens were in the form of solid state powders. They were put in the special powder cell holder. Before the measurements, correction of the baseline with a special calibration adapter was made. The accuracy of the apparatus was ±0.8 nm and the repeatability was ≤0.05 nm. The morphology of the materials was determined by scanning electron microscopy (SEM) using a LEO 1530 Gemini scanning electron microscope (Zeiss/LEO, Oberkochen, Germany). Prior to the analysis, the pigment powders were coated with a carbon target using a Cressington 208 HR system.

## 3. Results and Discussion

### 3.1. Secondary Ion Mass Spectrometry (TOF-SIMS) and ^27^Al NMR Analysis

**Figure 1:**
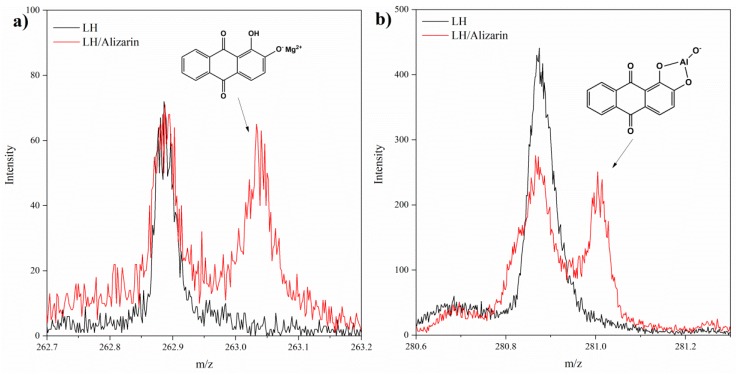
Positive (**a**) and negative (**b**) TOF-SIMS spectra of LH and LH/Alizarin (10%) samples.

The TOF-SIMS technique was employed to investigate the interactions between the matrix and the alizarin molecules. It was expected that alizarin would be stabilized in the LH matrix by ionic interactions with Mg^2+^ and/or Al^3+^ ions, and that such interactions would be revealed by the emission of characteristic ions from the LH/alizarin. This was confirmed by the presence of a C_14_H_7_O_4_Mg^+^ ion peak at m/z 263 in the TOF-SIMS spectrum of LH/Alizarin (Figure 1a). TOF-SIMS analysis was also used to investigate the formation of possible Al-alizarin complex ions. The presence of C_14_H_7_O_5_Al^−^ ions (m/z 281) may be ascribed to the fragmentation of complexes of alizarin with aluminum (Figure 1b). This is supported by the results of a study by Soubayrol et al. [24], in which it was found using NMR that alizarin formed a complex with Al^3+^ in both aqueous and methanolic solutions of sodium hydroxide. Complexes of alizarin have been reported with metals such as Ca or Mg [25]. However, the formation of complexes of organic chromophores with mixed oxides in LH may be hindered by steric limitations. This is due to the fact that aluminum-magnesium hydroxycarbonate consists of irregular plate layers. Alizarin molecules therefore have to be properly oriented towards the LH layers, in order to interact effectively with the Mg^2+^ and/or Al^3+^ ions. In traditional alizarin lakes, aluminum forms part of a chelate ring with the 9-carboxyl and 1-hydroxyl groups of alizarin, while a divalent cation (usually calcium) is bound as an ion [8]. However, in the current study, the formation of an alizarin lake with similar structure was impossible due to steric limitations, as the metal ions were trapped in the LH inorganic layers. It may be concluded that the color of the LH/Alizarin pigments was the result of the formation of individual alizarin complexes with Mg^2+^ and Al^3+^ ions.

**Figure 2:**
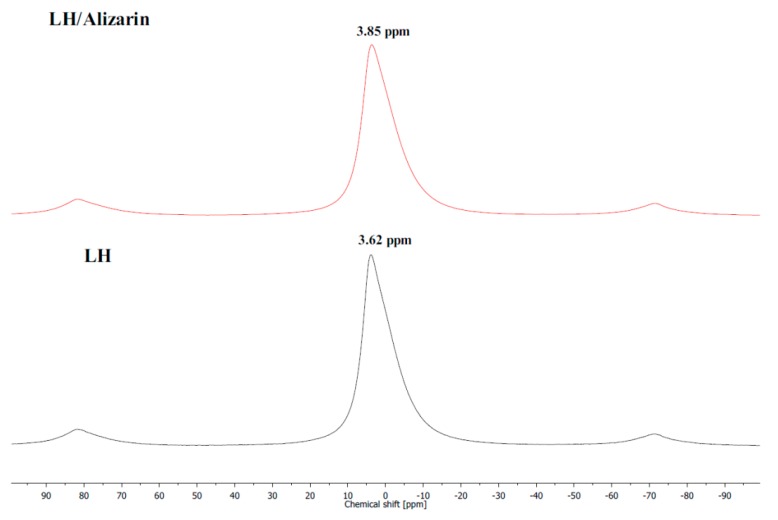
^27^Al MAS NMR spectra of alizarin lake before and after adsorption of alizarin dye.

Further analysis of the structure of the organic-inorganic framework was conducted using ^27^Al MAS NMR. ^27^Al resonance line positions are very sensitive to the coordination number and tend to occupy the −5 to 15 ppm range for AlO_6_ sites [26]. Figure 2 shows the spectrum of LH recorded before and after the adsorption of the anthraquinone dye. The resonance corresponding to hexacoordinated aluminum is clearly visible. The ^27^Al MAS spectrum of the LH sample before complexation shows a relatively narrow resonance peak at a chemical shift, δ, of ~3.6 ppm. The NMR signal of the hybrid pigment was different from that of the unmodified LH sample. Adding the organic chromophore to the LH host contributed to a chemical shift of the resonance peak from 3.6 to 3.9 ppm, which is very close to the chemical shift measured (3.4 to 3.2 ppm) for montmorillonite modified with alizarin in a reaction carried out under similar pH conditions (8.4) [21]. The results of NMR and TOF-SIMS provided complementary evidence of the interaction between the Al^3+^ ions and dye molecules.

**Figure 3:**
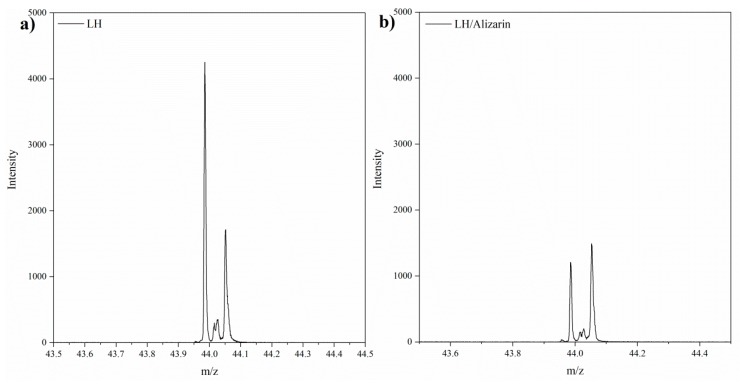
TOF-SIMS spectra of negative secondary CO_2_^−^ ions in LH (**a**) and LH/Alizarin (10%) (**b**).

The modification of the mixed oxide with alizarin was also accompanied by a reduction in the emission of CO_2_^−^ ions (Figure 3). This reduction was most probably related to the partial replacement in the LH host of CO_3_^2−^ ions by the organic chromophore, as a result of the complexation of C_14_H_7_O_4_^−^ ions with Mg^2+^ and Al^3+^ ions. This observation is in line with the results of further studies conducted using thermogravimetric analysis (TG). The mechanism for the formation of lake with aluminum-magnesium hydroxycarbonate seems similar to that for lake pigments obtained on traditional inorganic supports, involving interactions between the dye and relative insoluble alkaline earth salts [3,27]. It appears that CO_3_^2−^ ions from the weak carbonate acid are displaced from the edge of the LH interlayer by acidic dye molecules, forming insoluble alizarin pigments. A similar mechanism was posited in our previous work for alizarin lakes based on Mg-rich hosts [23].

### 3.2. Powder X-ray Diffraction (PXRD)

**Figure 4:**
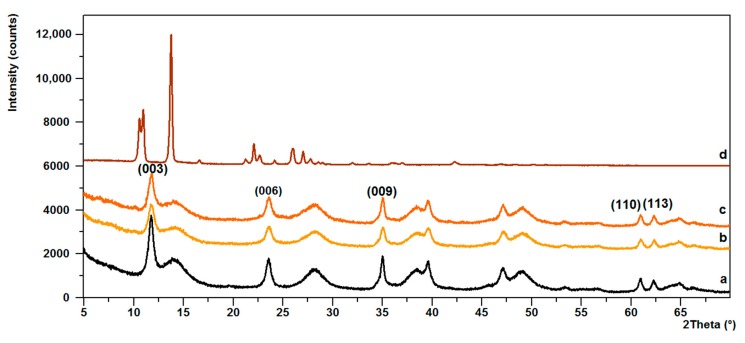
Powder XRD patterns for: LH (**a**), LH/Alizarin (15%) (**b**), LH/Alizarin (20%) (**c**), and alizarin (**d**).

Further structural analysis revealed that no changes had taken place in the basal spacing of the LH, following the arrangements of the alizarin dye in the mineral structure. The diffraction pattern of LH shown in Figure 4a consists of three sharp peaks at a low 2*θ* angle, equivalent to diffraction by planes (003), (006), and (009), respectively. This indicates the good crystallinity of the LH. The interlayer distances of (*d*_003_) and (*d*_006_), corresponding to 0.753 nm and 0.378 nm, are due to basal reflections indexed to a hexagonal crystal lattice with rhombohedral 3R symmetry [28]. Broadened peaks are observed on the diffraction pattern of the LH, which could be due to unaccounted for, poorly crystalline impurities. After the addition of alizarin, the basal reflection (003) of the LH/Alizarin pigments (Figure 4b,c) shifted slightly to lower 2*θ* angles, which for LH20, was 11.733° and for LH/Alizarine (20%), was 11.504°, although the interlayer distances remained similar, regardless of the content of dye molecules. The presence of a characteristic non-basal (110) reflection at 2*θ* ~60.90° confirms that the LH structure had been retained. The PXRD pattern for LH/Alizarin (20%) (Figure 4c) also shows very small reflections, characteristic of the crystalline structure of alizarin.

### 3.3. Thermogravimetric Analysis (TG)

**Figure 5:**
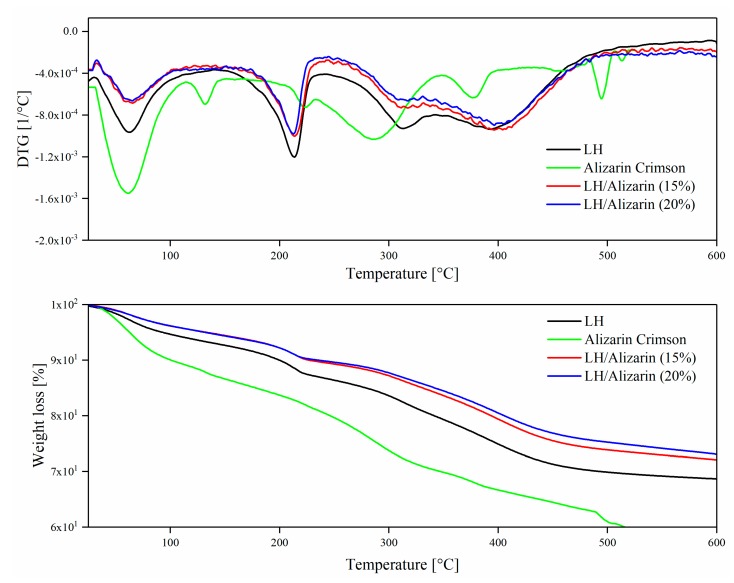
TG/DTG curves for alizarin lake with 10% and 20% alizarin content and Alizarin Crimson.

Thermogravimetric analysis (TG/DTG) was used to determine the thermal stability of the pigments. As shown in Figure 5, the thermal stability of the hybrid pigments improved significantly in comparison to the commercial alizarin lake and raw LH. The DTG curves of the hybrid pigments display multistep decomposition behavior typical of mixed oxides. The first stage below 100 °C corresponds partly to the desorption of water physisorbed on the external surface of LH, and partly to the removal of water molecules from the interlayer galleries. The second weight loss on the DTG curve with a strong peak centered at 213 °C can be attributed to the water produced by dehydroxylation of the LH layers. The final weight loss step occurs as the temperature increases, with two peaks at around 313 °C and 393 °C. The peaks can be attributed to the loss of interlayer carbonates and to the complete decomposition of metal hydroxide layers, respectively. It was during this stage that metal oxide structures formed [29]. The absence of a TG-DTG peak for the dye chromophore can be explained by the similar position of the dye degradation peak (326 °C) and the carbonate decomposition peak of the inorganic LH. However, the TG-DTG curve for Alizarin Crimson was shaped differently. Weight loss was observed at low temperatures (<200 °C), probably as a result of water loss. Above 200 °C, slow degradation was observed. The weight loss in the temperature range of 200–500 °C may be attributed to the degradation of the organic moiety.

As the concentration of alizarin increased, the thermal stability of the hybrid pigment improved gradually. After modification, the 10% weight loss temperature (T_10%_) of LH (199 °C) was increased to 227 °C for LH/Alizarin (15%) and to 235 °C for LH/Alizarin (20%) (Table 1). In comparison to Alizarin Crimson and unmodified LH, the hybrid pigments exhibited noticeably lower weight loss above 300 °C, due to the liberation of carbonate and hydroxyl groups from the layered mineral. Costa et al. [30] suggest that anthraquinone-like compounds can improve the thermal stability of LH systems, which may explain our results. On the other hand, the increased thermal resistance of the hybrid pigments may be the result of the strong incorporation of alizarin on the LH structure and the decrease in the concentration of carbonate ions, as confirmed by TOF-SIMS experiments.

### 3.4. UV-VIS Spectroscopy and Color Stability

**Figure 6:**
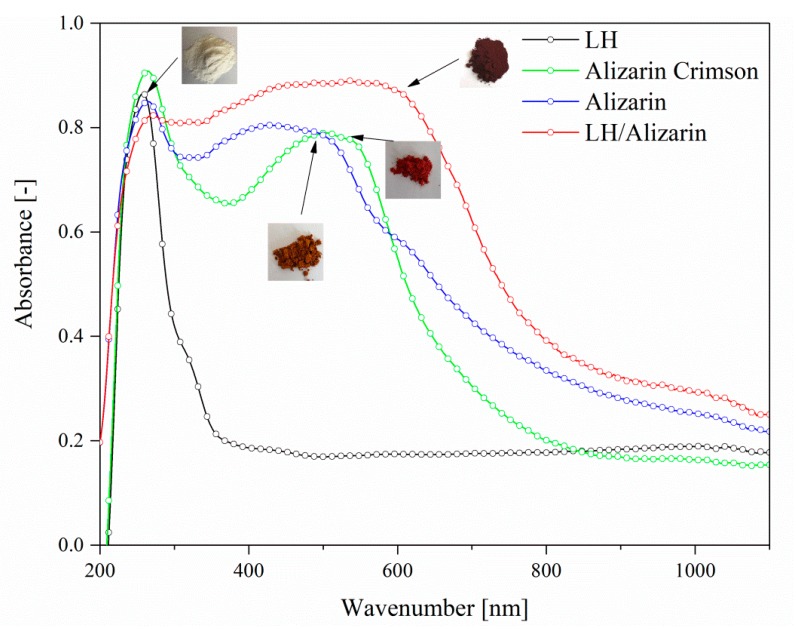
UV–Vis spectra of LH, alizarin, LH/Alizarin (10%), and Alizarin Crimson powders.

Figure 6 shows the absorption spectra for different LH, alizarin, Alizarin Crimson, and LH-based alizarin lake powders. The absorption spectrum of the alizarin chromophore shows a structured band with a maximum at 459 nm. The UV-Vis spectrum of the alizarin lake has a *λ*_max_ at 571 nm, with a significant bathochromic shift in comparison to free dye, due to complexation of the magnesium (II) and aluminum (III) ions into the LH layers. The variations in the color and hue of alizarin are pH-dependent (Figure 6) [21]. Neutral free alizarin can exist in several tautomeric forms. In solution, alizarin occurs in the form of partially dissociated yellow molecules below pH 5.2. At pH 6–10, it is deprotonated and occurs in red monovalent cations. Finally, at around pH 12, it occurs in violet di-anionic form. At low pH, the interactions between the organic dyes and the clay surface are generally driven by electrostatic forces. At a higher pH (such as in our study), interactions can occur via the complexation of chromophore structures with the metal cations present in the LH layers.

The pH of the reaction solution is not the only possible factor responsible for the variation in the optical properties of alizarin. The types of metals used can also have an effect on the color of the complexes they form with alizarin, as well as on their thermal and chemical stability. Complexes of alizarin with Al(III), Cr(III), Ni(II), Cu(II), Zn(II), Cd(II), and Fe(III)) are associated with a red-shift of the visible band with respect to the isolated dye [9,31,32]. Both environmental effects are present and influence madder lake color changes. The absorption band of the LH/Alizarin pigments in the visible region showed a maxim at higher wavelengths in comparison to a commercial lake, producing a dark red color with a violet hue. This result was in line with previous studies, in which alizarin complexes with metals such as Mg or Mn were associated with blue shades [9,23]. Therefore, the final color of the alizarin lake may be considered to be the result of the formation of individual alizarin complexes with both ions present in the LH structure (Mg^2+^ and Al^3+^).

**Figure 7:**
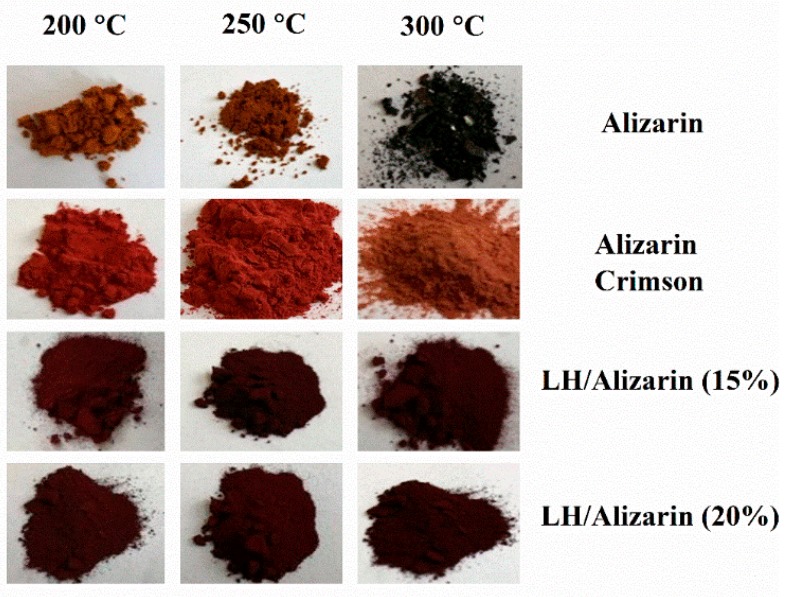
Color changes in alizarin, Alizarin Crimson, and alizarin pigments after thermal aging at different temperatures.

**Figure 8:**
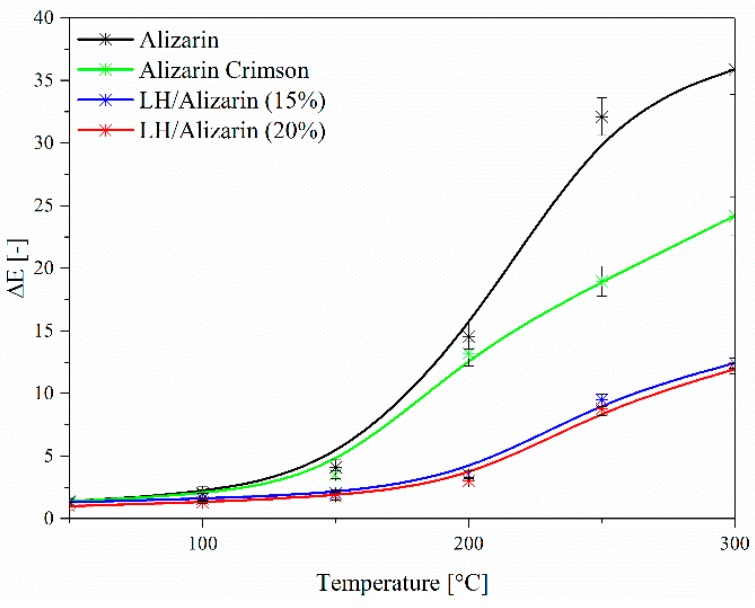
Color changes in alizarin, Alizarin Crimson, and alizarin pigments after thermal aging at different temperatures.

Figure 7 and Figure 8 present the digital photos and results of CIE Lab studies of alizarin, alizarin lake, and Alizarin Crimson powders, heated in an oven at 200, 250, and 300 °C, respectively, for 30 min. There were significant color differences (ΔE) in the samples thermally aged at 200 °C. As the temperature was increased, the changes in the total color difference parameter (ΔE) became much more marked, indicating that the alizarin chromophore and Alizarin Crimson began to decompose at 200 °C. In the case of the alizarin pigments, no significant variation in color was observed after heating at 200 °C.

**Figure 9:**
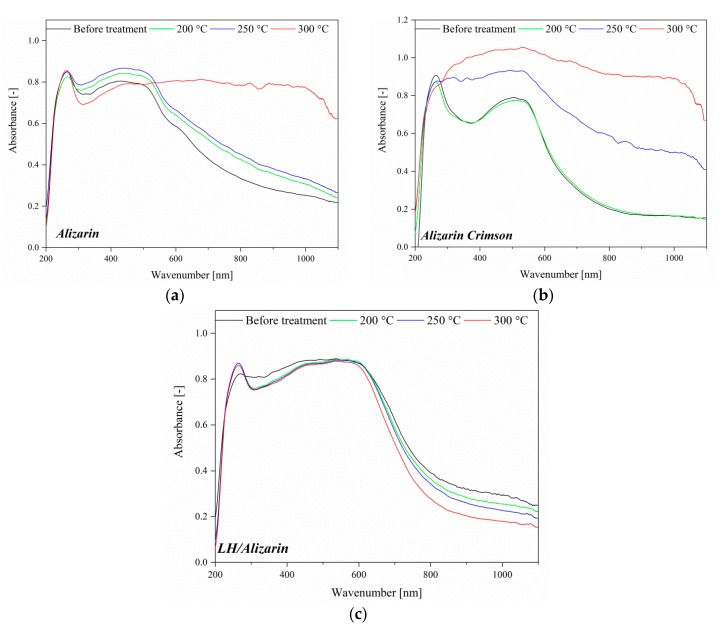
UV-VIS spectra of alizarin, Alizarin Crimson, and alizarin lake exposed to different temperatures: (**a**) alizarin; (**b**) Alizarin Crimson; (**c**) LH/Alizarin.

Some slight change in the color of the lake pigments was observed after 250 °C, resulting from the dehydroxylation of the LH layers. These results show that the alizarin lake can tolerate higher temperatures than pure chromophore or the commercial lake (Alizarin Crimson). The ΔE values for alizarin and Alizarin Crimson were larger than those of the LH-based lake after thermal aging above 200 °C, confirming that the LH/Alizarin pigments had a higher thermal stability than the reference samples. It can be concluded that the incorporation of alizarin dye into the LH structure significantly improved the thermal stability of the organic chromophore. This observation was confirmed by the diffuse reflectance UV-vis spectra of the studied pigments (Figure 9). The spectra of the dye and commercial lake showed marked changes after heating at 300 °C. However, in the case of alizarin pigments, there were no significant changes in the spectra after exposition at any of the considered temperatures.

### 3.5. Scanning Electron Microscopy (SEM)

**Figure 10:**
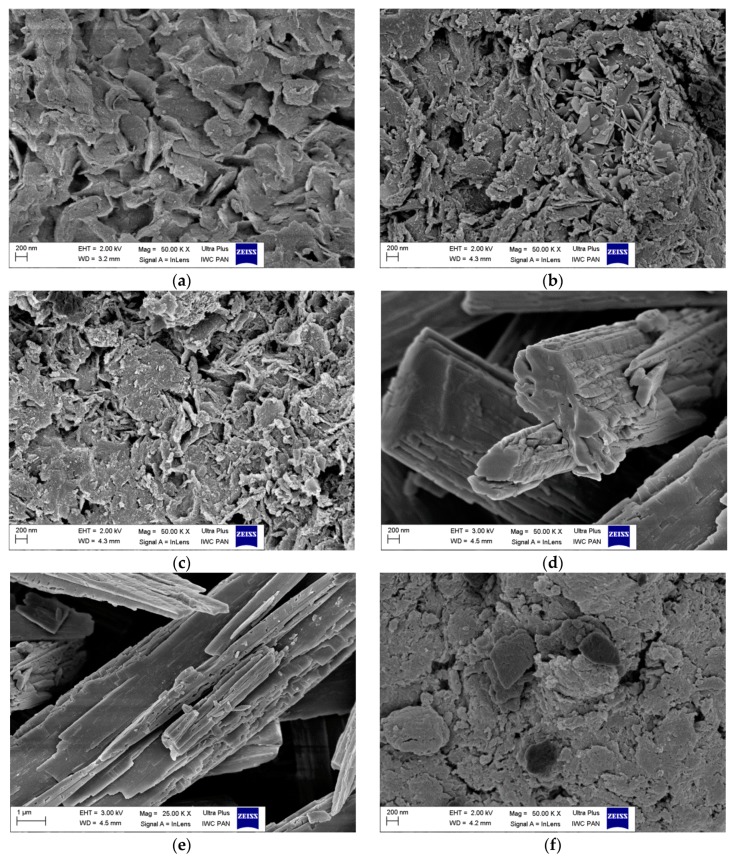
SEM images of LH (**a**); LH/Alizarin (15%) (**b**); LH/Alizarin (20%) (**c**); alizarin crystals (**d**,**e**); and Alizarin Crimson (**f**).

Scanning electron microscopy (SEM) experiments were performed to study the morphology of the hybrid pigments. The micrographs were obtained using 50,000× magnifications. As can be seen in Figure 10, the LH carrier was mainly composed of plate-like particles with thicknesses of a few hundreds of nanometers and a lateral dimension in the range of 400 nm to 1–2 µm. The particles had more or less regular structures and different degrees of sharpness around their edges. After modification, the structures of the hybrid pigments were quite similar to the structure of the parent LH, irrespective of the concentration of alizarin. However, the shapes of the particles were slightly more irregular than in the case of raw LH. Moreover, the new alizarin crystals appeared on the outer surface of the inorganic host, while the layered structure of the LH remained unchanged. Interestingly, the structure of the new crystals on the LH differed significantly in comparison to their initial states. Originally, the alizarin took the form of oblong block/needle-like shape crystals, while after modification, some irregular crystals appeared. In the case of the commercial lake, completely different morphological features were observed. Agglomerations of particles with more irregular structures formed. Scanning electron microscopy thus enabled the study of both the morphology and the aggregation of the particles.

In general, HT-like compounds are known for their relatively low specific surface areas. Due to their lack of layer ordering and irregular shapes, the particles of LH exhibited a higher surface area value of 201.4 ± 17.2 m^2^/g in comparison to LH with a higher concentration of magnesium ions, as measured in our previous work (107.4 ± 9.5 m^2^/g) [23]. As anticipated, after modification with 15% and 20% alizarin, the surface area of the LH decreased to 157.2 ± 13.3 m^2^/g and 125.4 ± 11.7 m^2^/g in each respective case.

### 3.6. Solvent Resistance

The stability of the prepared hybrid pigments was evaluated based on their resistance to selected organic solvents and water. Since anthraquinone dyes are highly soluble, sustained washing with water and/or organic solvents should remove any excess or weakly physisorbed dye from the LH surface. The degree of discoloration after 24 h of exposure to solvents was therefore assessed, on a scale of 1/5 (where 5 means total insolubility).

**Figure 11:**
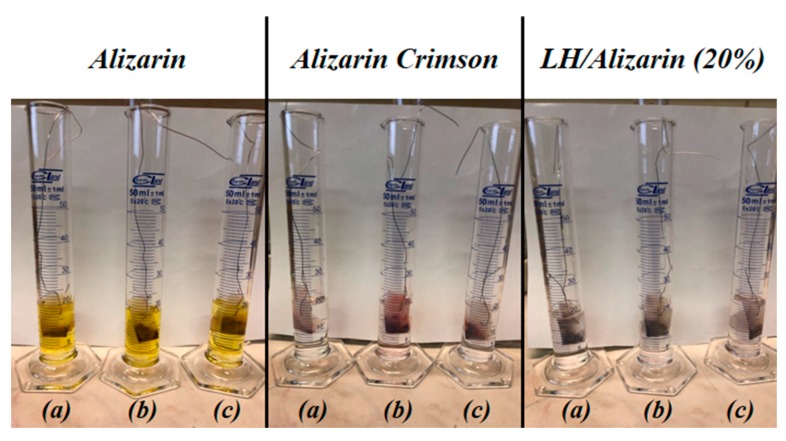
Digital images of alizarin, Alizarin Crimson, and alizarin pigments after 24 h of immersion in toluene **(a)**, acetone (**b**), and ethyl alcohol (**c**).

Based on the experiment, it can be concluded that both Alizarin Crimson and the alizarin pigments exhibited excellent solvent resistance, as the established values for all media were 5. Moreover, from Figure 11, it can be seen that the solvents containing the unmodified alizarin chromophore had a very intense yellow color, whereas the solutions with the hybrid pigment and Alizarin Crimson were colorless. This confirms that the alizarin molecules had been effectively stabilized onto the aluminum-magnesium hydroxycarbonate surface, transforming the soluble dye into a solvent-resistant hybrid pigment. It is also worth noting that the hybrid pigments with both 15% and 20% concentrations of the chromophore were characterized by an equally good resistance to organic solvents. The LH host produced alizarin pigments with a higher solvent resistance than matrices with a higher concentration of magnesium, as had been used in a previous study [23]. This is most likely related to the aforementioned difference in the surface area values of the inorganic matrices.

## 4. Conclusions

In this study, we investigated the possibility of stabilizing alizarin on solid state mixed oxides. A new type of hybrid pigment was created by the complexation of alizarin dye with an aluminum-magnesium hydroxycarbonate inorganic host. ^27^Al solid-state NMR and TOF-SIMS were applied to study the local structures of pristine LH and the chemically modified LH matrix. The dye molecule interacted with different ions simultaneously and the final color was a result of the combination of different alizarin complexes. The stability of the hybrid pigments was tested under solvent and temperature treatment. At both 15% and 20% dye concentrations, the metal-dye complexes showed excellent resistance to dissolution in solvents: acetone, ethyl alcohol, and toluene. A further advantage was found to be their considerably improved thermal and color stability in comparison to the commercial lake. These enhanced properties can be explained by the strong affinity of alizarin to LH, which leads to the formation of stable complexes with Al^3+^ and Mg^2+^ ions. TOF-SIMS proved to be a powerful method for studying the interactions in solid state materials. TOF-SIMS can be considered as a complementary technique alongside NMR, providing insight into the mechanisms by which anthraquinone chromophores are stabilized by a mixed oxide host.

## Figures and Tables

**Table 1:** Thermogravimetric analysis of LH/Alizarin pigments.

Sample	^1^ T_05%_ (°C)	T_10%_ (°C)	T_20%_ (°C)	T_30%_ (°C)
LH	93	199	342	493
Alizarin Crimson	72	120	301	427
LH/Alizarin (15%)	133	227	393	616
LH/Alizarin (20%)	132	235	406	620

^1^ T_05_, T_10_, T_20_, T_30_—Temperature of 5%, 10%, 20%, and 30% weight loss, respectively.
